# Adjustment Disorder and Suicidal Behaviours Presenting in the General Medical Setting: A Systematic Review

**DOI:** 10.3390/ijerph16162967

**Published:** 2019-08-18

**Authors:** Joanne Fegan, Anne M. Doherty

**Affiliations:** Department of Psychiatry, University Hospital Galway, H91 YR71 Galway, Ireland

**Keywords:** adjustment disorder, depressive episode, self-injurious behaviour, liaison psychiatry, diagnosis, suicide/attempted

## Abstract

*Background:* Adjustment disorder (AD) is a condition commonly encountered by clinicians in emergency departments and liaison psychiatry settings and has been frequently reported among patients presenting with suicidal behaviours. A number of previous studies have noted the strong association between suicidal ideation and behaviours, and AD. In this paper, we aimed to explore this relationship, by establishing the incidence of AD in patients who present with self-harm and suicidal ideation, and the rates of self-harm among patients with a diagnosis of AD. *Methods:* We conducted a review of the literature of well-established databases using specific key words then synthesised the results into a descriptive narrative as well as representing it in table form. *Results:* Sample sizes and study methods varied significantly across the review. A majority of studies were retrospective chart-based reviews, and only three used structured diagnostic instruments. A high prevalence of AD (ranging from 9.8 to 100%) was found, with self-poisoning representing the most common form of suicide attempt in the majority of studies. Interpersonal difficulties were the main precipitant in studies which examined this. *Conclusions:* This study suggests there is a strong association between AD and suicidal behaviours. Given the paucity of research in the area, there is a need to build the evidence base for effective treatment strategies.

## 1. Introduction

Adjustment disorder (AD) is a condition which is characterised by the development of symptoms, usually of depression or anxiety, in response to a stressful event [1]. This condition is frequently diagnosed in patients attending Emergency Departments (EDs) and liaison psychiatry settings, and in particular, has been commonly reported among patients presenting with suicidal behaviours, including self-harm. 

Suicidal ideation and behaviours may be a feature of a number of psychiatric disorders and are an important symptom, indeed diagnostic criterion, in depression [2]. AD is defined by the World Health Organisation in the International Classification of Diseases, 10th Edition (ICD-10) as a state of “subjective distress and emotional disturbance, usually interfering with social functioning and performance, and arising in the period of adaptation to a significant life change or to the consequences of a stressful life event” [2]. A diagnosis of AD requires the identification of a precipitating stressor, and symptoms must resolve within six months of the termination of the stressor. This diagnosis occurs where the symptoms are not more appropriately attributed to another mental disorder. The ICD-10 diagnostic criteria do not specify the symptoms of AD beyond “those found in any of the affective disorders”. However, some indications of typical symptoms are suggested by the subcategories of AD in ICD-10, which include “brief depressive reaction”, “prolonged depressive reaction”, “mixed anxiety and depressive reaction”, indicating the common presentations of the condition [2]. Similarly, the American Psychiatric Association’s classifcation system, the Diagnostic and Statistical Manual of Mental Disorders, 5th Edition (DSM-5) categorises AD as presenting: “with depressed mood”, “with anxiety” and “with mixed anxiety and depressed mood” [3], and has symptomatic overlap with depression and anxiety. 

AD has been described as a controversial disorder [4], especially with respect to its role in the classifications systems. The key characteristics of AD have remained stable since it was first described in the diagnostic classification systems, and include symptoms common to both depressive and anxiety disorders. Although, unlike depressive episode, there is no prescribed list of clinical symptoms required for the diagnosis of AD in ICD-10 and DSM-5, and there may be significant clinical overlap between the two conditions in terms of symptomatology [5,6]. Some symptoms, including arguably the most severe and life-threatening symptom, suicidal ideation, may be as common in AD as it is in depressive episode [7]. 

The proposed ICD-11 will re-categorise AD under conditions specifically caused by stress, along with other conditions such as post-traumatic stress disorder. It will define the diagnosis of AD in a more positive manner, in describing two core symptoms: (a) Preoccupation with the stressor, and (b) failure to adapt. Preoccupation with a stressor includes recurring distressing thoughts or ruminations on the theme of the stressful situation, while failure to adapt is a more general difficulty, which brings as a consequence of the preoccupation disturbance including those of sleep and concentration resulting in an impairment of function across key domains, such as social or occupational functioning [8].

AD has not been included in the major epidemiological studies of mental disorders, and as a consequence, the incidence and prevalence rates in the general population are unknown. The two clinical areas which have come closest to providing epidemiological data on this condition are general practice and liaison psychiatry. Huyse et al., in a European study of fifty-six consultation-liaison psychiatry services in eleven countries found that adjustment disorder accounted for a significant proportion of psychiatry morbidity in acute medical hospitals [9]. While self-harm, at 17%, was the most common reason for referral, adjustment disorder and post-traumatic stress disorder was diagnosed in 12.4% of those patients referred. Unfortunately, this paper did not examine the relationship between self-harm and diagnosis [9]. 

Our hypothesis is that AD is a common disorder in patients who present to emergency departments of hospitals with suicidal ideation and behaviours, i.e., present for assessment by liaison psychiatry services.

In this study we aimed to examine the association between suicidal ideation and behaviours in AD in an acute medical hospital setting. The objective of this study was to establish the incidence of AD in patients who present with self-harm and suicidal ideation, and the rates of suicidal ideation and behaviours among patients with a diagnosis of AD.

## 2. Materials and Methods

A comprehensive search strategy was developed and used to search electronic databases (PubMed, CINAHL, Medline, Psychinfo and the Cochrane Library) for published studies on suicidal behaviours in adjustment disorders using the search terms, “adjustment disorder”, “suicide”, “adjustment disorder AND suicide”, “adjustment disorder AND overdose”, “adjustment disorder AND self-harm”, “adjustment disorder AND suicidal ideation and behaviours” and “adjustment disorder AND general hospital psychiatry”. The search was confined to material published within the last thirty-five years. A further filter requiring the published articles to be peer reviewed was also applied. Studies written in a language other than English studies were excluded, in addition to letters, editorials, commentaries and textbooks.

We included studies which met the following criteria:

(a) Included patients diagnosed with AD, either clinical diagnosis or using structured diagnostic instruments.
(b) Conducted in medical settings where the specialty of liaison psychiatry is to be found i.e., emergency departments/rooms, general medical wards, critical care units etc.
(c) Described patients presenting with self-harm (regardless of suicidal intent) or suicidal ideation.
(d) Studies that included at least one clinical characteristic in addition to diagnosis (self-harm methods, previous attempts, etc.
(e) Observational studies with or without comparison groups.

The exclusion criteria were review papers, letters, editorials, commentaries, abstracts for which there were no data available. We excluded all studies from the non-liaison psychiatry population, i.e., those who recruited from anywhere other than a general medical setting, where there was specialist psychiatry input.

The study selection process was conducted in the first instance by one reviewer (JF) and independently validated by a second reviewer (AMD). A meta-analysis of data was planned, but could not be performed due to inherent heterogeneity in the studies. This heterogeneity may explain why no previous meta-analysis of this kind was identified in the Cochrane Library. 

For rating the methodological quality of the included studies, this study used the Quality Assessment Tool for Observational, Cohort, and Cross-Sectional Studies of the National Institutes of Health (NIH) [10], as modified by Troya [11]. For each study, the quality was assessed independently by the reviewers separately, to give a rating of high, moderate or poor to each study.

Results of the review were synthesised into a descriptive narrative under specific headings highlighting the prevalence of AD, the demographic profile and the suicide methods used, and were also summarised in a descriptive table (Table 1). 

## 3. Results

**Figure 1:**
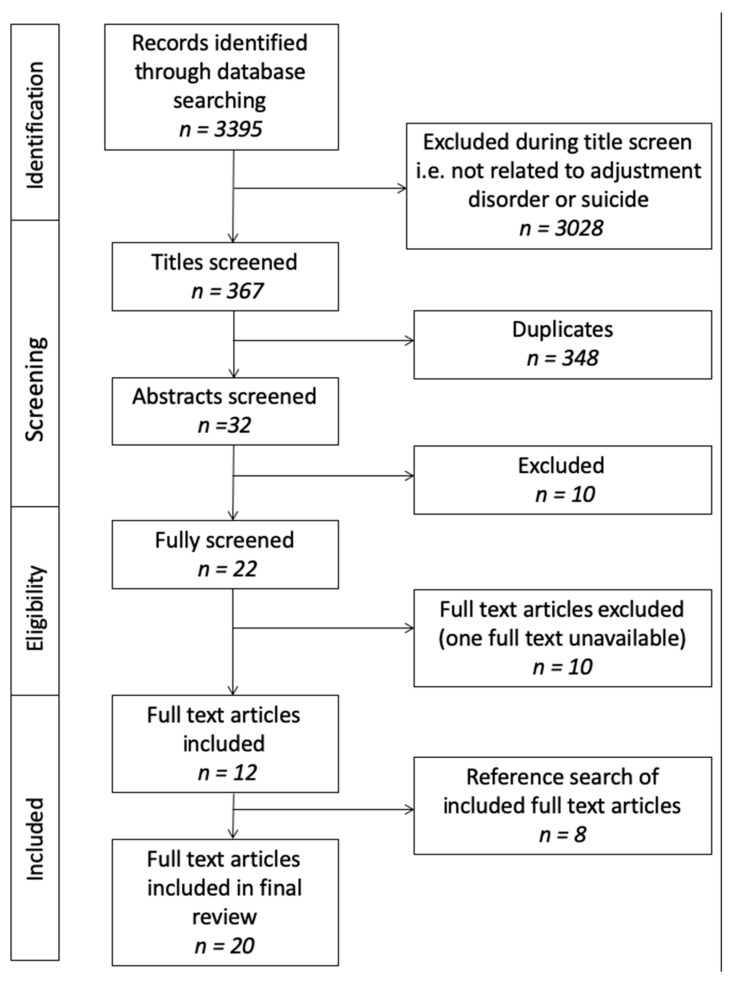
Study flow diagram.

The initial search yielded 3395 articles. Of these, 348 articles were identified as duplicates and discarded. A further 3028 were excluded during the title screen as they were not related to AD or to suicidal behaviours. The remaining 32 articles were screened by abstract, at which stage 10 of these were excluded, leaving 22 articles for the full text screening process. A further 10 articles were excluded, with 12 studies remaining from the search. A further 8 studies were found by hand-searching the references of the included studies. A total of 20 full text articles were included in the final review (Figure 1).

### 3.1. Description of Studies

The 20 included studies were all from a general hospital setting, including EDs (*n* = 11; 55%), medical wards (*n* = 3, 15%) or specialised toxicology/burns units (*n* = 2; 10%). The remainder (*n* = 4; 20%) included patients across these settings. Over half of the studies were from countries where English is not the first language (*n* = 11; 55%) with 40% (*n* = 8) from English-speaking countries and 1 study from a number of English-speaking and non-English speaking countries. Half (*n* = 10; 50%) were retrospective chart reviews, and the remainder either cross-sectional (*n* = 3; 15%) or prospective cohort studies (*n* = 7; 35%). The studies are further described in Table 1. 

### 3.2. Methodological Quality of Studies

**Figure 2:**
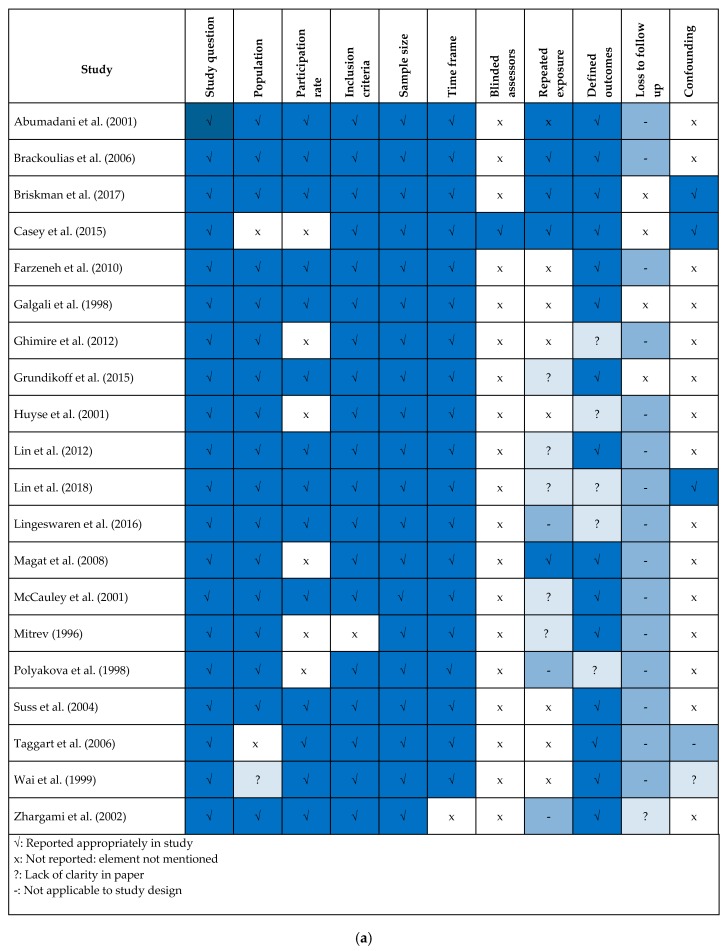

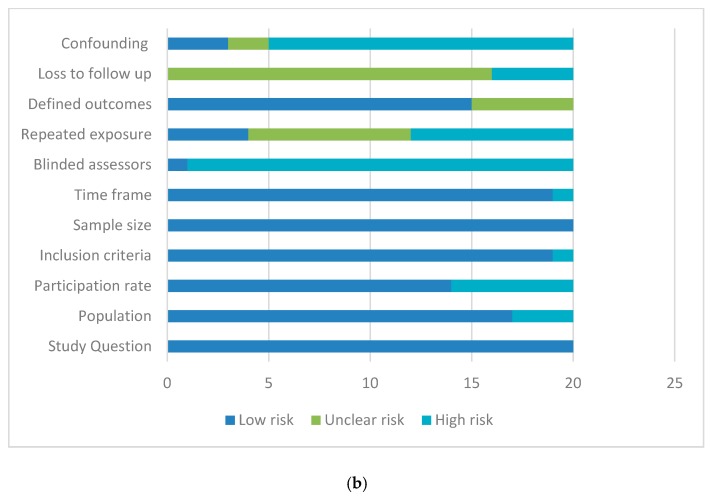
(**a**) Quality assessment within studies, (**b**) Quality assessment across studies.

The 19 included studies were assessed using the NIH Quality Assessment Tool for Observational, Cohort, and Cross-Sectional Studies [10]. Figure 2a provides an overview of the quality of the included studies, and Figure 2b highlights the areas of risk across the included studies as a whole, grouping as high, low or unclear risk. Identified high risk areas ≥80% included measure of and adjustment for key confounding variables and blinding of assessors. Low risk areas ≥60% included clear elucidation of the study question, clear specification of the population studied, clearly prespecified inclusion criteria, sample size description and description of time frame. Areas of unclear risk ≥60% included loss to follow up.

### 3.3. Socio-Demographic Factors

#### 3.3.1. Age

Four of the twenty (20%) studies included, focused exclusively on paediatric populations [12,13,14], and eight (40%) had a mixed demographic which included those under the age of eighteen as well as adults. Of these Lingeswaran et al. described a population with age range of ten to thirty years [15]; Mitrev reported an age range of fifteen to over sixty years [16], and Ghimire et al. described a population of two hundred presentations to an ED in Nepal that ranged in age from fifteen to fifty-five years (77% of whom were aged under 34 years) [17]; McCauley et al. described a population aged between 10 and 60 years in an ED in a hospital in rural Ireland, close to half of the sample were aged less than thirty [18]; Galgali et al. reported a mean age of 25 (SD = 8.1) in their sample, without describing a range [19]; Zarghami et al. reported an average age of 27 years (SD = 13.5), again without specifying the age range [20]; Abumadani et al. report an patients in Saudi Arabia aged 13–74 [21] and Wai et al. focused on a young adult/adolescent population all aged under 21 [22]. 

Four studies (22.2%) focused exclusively on an adult population, Casey et al. reported a mean age of 36.5 years (SD = 10.1) in those presenting with AD and suicidal behaviours in three Dublin hospitals [23], Polyakova recruited only patients aged over 18–65 years to their ED-based study of AD and self-harm [7]. Brakoulias, in a larger study of self-harm presentations to a liaison psychiatry service in Australia included adults only aged 18–88 years [24]. Briskman examined patients presenting with self-harm aged over 18, comparing those aged over and below 65 years, the only included study that specifically examined older patients [25]. The remaining four studies did not specify the age range of participants.

#### 3.3.2. Gender

Eighteen of the twenty studies (90%) showed a higher proportion of females than males in suicidal populations, with five studies having women representing more than 80% of the sample: 86% female in Magat et al.’s study, 83% in Zhargami et al., 81.5% in Suss et al., 80.4% in Farzeneh et al. and 80% in Abumadani et al. [12,13,14,20,21]. The two studies with a majority of male participants were both from Taiwan: with 60% male participants in Lin’s 2012 study of charcoal poisoning, and 75% male in Lin’s 2018 study of rodenticide and paraquat poisoning (78% in the paraquat subgroup were male) [26,27]. 

### 3.4. Frequency of Adjustment Disorder (AD) Diagnosis

The majority of the studies used a clinical diagnosis rather than a diagnosis based on a semi-structured interview: Only three studies (15.7%) used a semi-structured interview. Zhargami et al. used the Structured Clinical Interview DSM version 1 (SCID-I) a diagnostic semi-structured interview based on DSM-III [20]. Taggart et al. also used SCID, and noted a significant difference in the rates of diagnosis depending on whether clinical diagnosis or SCID diagnosis was used [28]. This study reported rates of AD of 32% when using clinical diagnosis, and 7.8% when using SCID. Casey et al. similarly used both the Schedule for Clinical Assessment in Neuropsychiatry (SCAN) a diagnostic semi-structured interview based on ICD-10, and clinical diagnosis [23]. Casey et al. ultimately reported on the clinical diagnosis rather than the SCAN diagnosis, noting the inherent weaknesses in the semi-structured schedules in the diagnosis of AD [23].

Three studies specifically selected patients with AD, the remainder examined a more general cohort of patients presenting with suicidal ideation and behaviours. Mitrev’s study selected only patients with AD attending a toxicology unit for emergency treatment of self-poisoning, and examined the characteristics of these patients in terms of ongoing suicidal risk—they found a significantly higher risk in those with chronic AD and pervious suicidal behaviours [16]. Polyakova and Casey both selected patients with AD and compared them to patients with major depression [7,23]. Polyakova’s study of 155 participants recruited from a Moscow ED had 55.5% AD and 44.5% depression [7]. Casey’s study recruited 348 patients from three Dublin hospitals: 49.7% of whom had a clinical diagnosis of AD, and 50.3% depression [23]. 

The remaining studies reported the percentage of patients with a diagnosis of AD where the researchers were not specifically recruiting this diagnosis. A number of the studies reviewed found AD to be a common diagnosis among those presenting for emergency assessment following self-harm. The lowest proportion of AD found was reported by Lin et al. (9.8%) in patients presenting with self-poisoning with either rodenticide or paraquat in Taiwan [27]. Ghimire et al. reported that 13.5% of the patients presenting to a Nepalese ED with self-poisoning had a clinical diagnosis of AD [17]. Magat reported AD in 29% of patients presenting for treatment following self-poisoning in Hawaii [13]. Abumadani et al. found AD was the clinical diagnosis in 30.1% of their study population [21]. Taggart at al. examined patients who presented to emergency departments in Belfast following self-harm and found AD (31.8%) was 1.5 times as common as depression (19.5%) [28]. Galgali’s ED based study, where ingestion of pesticides was the most common form of self-injury, found 33.7% of the 119 cases of attempted suicides, referred for psychiatric assessment over a 12 month period, received a diagnosis of AD—the most common diagnosis in this study [19]. AD was the most common diagnosis in McCauley et al.’s study of self-harm in Ireland at 35.8% [18]. 

An Australian study of emergency referrals found that 35.9% of referrals to a new psychiatric Emergency Care Centre in Sydney had a diagnosis of AD; furthermore, AD was the most common diagnosis in those presenting with suicidal behaviours [24]. A 10-year retrospective study of attempted suicide by charcoal burning in Taiwan, where this is a common method of suicide, found that 41% of people presenting with attempted suicide by this method met the diagnostic criteria for a diagnosis of AD [26]. Zhargami et al., in a study based in a burns unit in Iran found that 42.1% of patients referred with self-immolation had a clinical diagnosis of AD [20]. 

Wai et al. reported a diagnosis of AD in 53.5% of patients presenting with self-injury to an ED in Singapore [22]. Suss et al. reported AD in 77% of serious or high-risk suicide attempts, and 50% of the lower-risk attempts [14]. Farzaneh et al. found 80% of a population of students presenting with self-poisoning to a specialist poison centre in Tehran over a year, had a diagnosis of AD [12]. Lingeswaren et al. reported that 100% of the people seen with self-poisoning had a diagnosis of AD. It is not clear from the study whether a diagnosis of AD was a selection criterion or whether all patients who presented during the timeframe of the studies happened to have a diagnosis of AD [15].

### 3.5. Suicide Attempt Method and Mortality Rates

The majority (eighteen) of the twenty studies in this review examine cohorts who have presented with suicidal behaviours. Casey et al., and Grundikoff et al. both report on individuals presenting with suicidal behaviours as well as suicidal ideation [23,29]. Lingeswaran et al. had death as an exclusion criterion. This study reported that 60% of the sample acted impulsively i.e., within thirty minutes of the suicidal thought and that interestingly, 97% had no previous attempt or family history of suicide [15]. 

Only two studies reported on patients who died by suicide following their presentation with self-injury. As a result, this study cannot comment on mortality rates in this population. Zarghami et al. reported that 79% of the cohort had died as a result of self-immolation [20], and Lin et al. reported in their study of poisoning by rodenticide and paraquat that 50% of the cohort (all in the paraquat group) died [27]. 

Overall, the most common form of suicide attempt was self-poisoning, the sole means of attempt in almost half of the included studies (*n* = 8; 40%), and the most common method in a further seven studies: 70%, 78.7%, 79.8%, 83.2%, 90%, 92.5% and 92.9%, respectively [7,18,21,22,24,28,29]. Suss et al. examined adolescents attending a New York ED for treatment of non-fatal overdoses. They found that the majority (77%) of the more serious suicide attempts received a diagnosis of AD from a consultant psychiatrist [14]. Grundikoff et al. did not provide any detail on the suicidal behaviours in the paediatric population studied [29].

### 3.6. Precipitants

Casey et al. compared suicidality in two groups, one with a diagnosis of AD and the other with a depressive episode. They found that those with AD experienced more life events, higher rates of personality disorders and higher rates of suicidal behaviours at a younger age and a lower depressive symptom threshold, than those with a depressive episode. The possible role of personality disorder in this finding was insignificant on multivariable analysis [23]. Farzeneh et al. found that almost a third reported romantic disappointment as the main reason for attempting suicide whilst more than half claimed family conflict [12]. A 1998 study of 308 people presenting with self-poisoning to a hospital in Bangalore, found that more than a quarter cited problems within their primary support group (26%) as the main stressor, whilst 58% had no identifiable trigger for their suicide attempt [19]. The most common precipitant of the suicidal act in Mitrev et al.’s study, was problems in the primary support group (in most cases, family) which was reported in 98 (70%) of the 140 cases [16]. Wai et al. found that 24.5% of patients who attended an ED in Singapore after a suicide attempt cited conflict with family as their suicidal trigger whilst a further 23.6% alluded to conflict with friends [22]. Magat et al. found that 22% of those attending an ED in Hawaii had had an argument with a family member whilst 11% had experienced conflict with a significant other prior to a suicide attempt [13]. Ghimire et al. make a distinction between interpersonal conflict and conflict within a marriage, and found that 72% of the cohort (*n* = 200) presenting for medical treatment for deliberate self-harm, identified interpersonal conflict as the trigger for suicidal behaviour, whilst 14.5% cited marital conflict. A further 3.5% claimed romantic disappointment [17]. Grundikoff et al. reported family conflict in 41% and peer conflict in 20.4% of the patients presenting with suicidal behaviours in the paediatric population studied [29].

## 4. Discussion

AD is a common condition among patients presenting for treatment following suicidal behaviours, across the studies where it is recorded as a diagnosis. AD is diagnosed with high frequency in suicidal populations across multiple studies in differing nationalities and ethnic groups (Table 1). AD was the exclusive diagnosis in two of the studies: Lingeswaran et al., retrospectively examined case notes of adolescents presenting to an emergency department in India for treatment post self-poisoning [15], and Mitrev examined 140 patients attending a toxicology centre in Germany after self-poisoning in a prospective study [16]. A majority of the included studies were retrospective reviews of case-notes. This methodology brings with it some biases (selection bias, information bias). 

The rates of diagnosis of AD among individuals presenting with self-harm are not dissimilar to the rates reported in psychological autopsy studies, although are lower on average. Portzky et al., in a psychological autopsy study in Belgium, found AD to be the second most common diagnosis in this group, accounting for 21.1% [30]. Likewise, Martunnen found that 21% of adolescent deaths by suicide were related to a likely diagnosis of AD [31]. Lin’s national database study of Taiwanese people admitted to medical and psychiatric hospitals with self-harm (*n* = 57,874) reported that AD is associated with a significantly increased risk of repeated suicidal behaviours (OR 1.8) but a significantly reduced risk of death by suicide (OR 0.12) [32].

The studies included in this review focused on a variety of age groups. From children and adolescents only, adults only to the whole range of ages presenting with suicidal behaviours, with a number of studies not identifying the age range included. The data included here suggests that AD is an important diagnosis in young people and one that is associated with severe symptoms. Most of the studies reported a majority of females presenting with suicidal behaviours. 

In most studies included, the most common form of suicidal behaviour reported was self-poisoning, with three-quarters (*n* = 15; 75%) of studies reporting that >70% of participants used this means. Triggers or precipitants were varied, but interpersonal difficulties in various forms including family and romantic were commonly reported as the precipitating stressors. Similarly, in a study of adolescent inpatients in psychiatric hospitals, Chiou et al., found 25% of those who had attempted suicide cited conflict with a parent as the main precipitant to suicidal behaviour, whilst 10% reported interpersonal difficulty either within a romantic relationship or with a friend as the main stressor [33]. A systematic review described psychological pain as a key factor identified in the suicide notes of people who died by suicide [34]. The findings of this study, and in psychological autopsy studies suggest that there may be overlap between this psychological pain and the diagnosis of AD, which is characterised by significant distress regarding one or more stressors (causing psychological pain).

Perhaps the most striking finding of this paper is the small number of studies (of the great many which have examined suicidal behaviours) which have included AD as a diagnosis. This may be related to the inherent difficulties in diagnosing AD when relying on structured interviews, many of which only include AD in an appendix, only to be used if the threshold for another disorder cannot be met. This approach, ignoring context, has been criticised by many researchers in the area of stress-related disorders [5,35]. The majority of the studies included have used clinical diagnosis, and as a result have utilised the clinician’s clinical judgment about the role of context and stressors in the patients’ presentations. This might be perceived as a weakness of the included studies, but given the controversy around AD and its diagnosis using structured tools leading to researchers describing clinical diagnosis for all its faults as the “gold standard”, it can be argued that this is instead a strength of these studies [36]. In just two of the included studies, both from Ireland by Taggart et al. and Casey et al., clinical diagnosis and semi-structured clinical interview schedules were used. In both cases the semi-structured clinical interview schedules diagnosed depressive episode, where the clinical diagnosis was AD [23,28]. A possible solution to this difficulty has been presented by the new classification system of ICD-11, which gives a clearer framework to allow a diagnosis of adjustment to be made using positive symptoms and accounting for clinical context [8]. This will allow future diagnostic schedules to include AD in a more consistent and reproducible manner, and will strengthen the research in this area.

### 4.1. Strengths and Limitations

This is the first systematic review of the association between the diagnosis of AD, and suicidal ideation and behaviour, and encompasses all the literature published in the area as identified by the literature search.

The conclusions of this study are limited by the paucity of research in the area. We identified 14 studies, most of which were observational studies of small numbers of patients—the largest being 348 patients. 

Another limitation to this study is absence of data in most of the studies of the degree of suicidal ideation or intent underpinning the suicidal presentations. This is also related to the methodology of retrospective review, used in the majority of the studies.

### 4.2. Further Research

This study identifies the need for further research into both AD as a diagnosis and into the association of this diagnosis with suicidal behaviours. This systematic review suggests that there is a strong association between suicidal ideations and behaviours and AD, especially in the general hospital setting.

## 5. Conclusions

This study confirms the association of AD with suicidal ideation and behaviours in multiple countries and once more highlights the increased risk in young adults, particularly females. Given the high representation of self-poisoning as a method of suicide attempt, future public health campaigns may need to consider stricter controls on over the counter medications and education of populations regarding safer practices around storage of potentially toxic compounds like pesticides. AD represents an important disorder to target in suicide prevention initiatives.

## Figures and Tables

**Table 1:** Characteristics of included studies (*n* = 20).

Study	Type	No. of Participants	Setting	Age	Diagnosis	Study Length	Self-Harm Method	Previous or Subsequent Attempt	Death	Influencing Factors/Precipitants
AbuMadidi et al. (2001)	Retrospective study (chart review)	398	ED, Saudi Arabia	13–74 years	AD 30.1%; personality disorder 32%, depression 8.6%	6 years	78.7% poisoning; 26% cutting	Previous attempt in 21.5%	Not stated	Females more likely to have dx AD (*p* < 0.01), stressful life events (*p* < 0.001). Males more likely to have substance misuse (*p* < 0.001), psychosis (*p* < 0.01)
Brakoulias et al. (2006)	Retrospective study (chart review)	1295	Liaison psychiatry service, Australia	18–88 years	AD 35.9%; major depression %; schizophrenia	5 years	79.2% poisoning; 12.7% cutting; 4.7% violent	12% prior self-harm	Not stated	Women more likely to poison, men more likely to cut or violent act. Separated and divorced women 18–24 high risk. Violent group, AD less common than depression or schizophrenia.
Briskman et al. (2017)	Prospective cohort study	1149	ED Israel	18–95 years	AD 48% most common diagnosis, personality 16%; depression; 15% depression	8 years	92.5% poisoning; 7.5% hanging; cutting	30%	Not stated	AD 46.7% aged 16–64, 57.8% in >65 years
Casey et al., (2015)	Prospective cohort study	348	Liaison psychiatry services in 3 Dublin Hospitals	Mean age in AD with suicidal behaviour 36.5 years	AD 49.7%; depressive episode 51.3%	6 months	Not stated	Not stated	None	Younger age, single marital status and greater severity of depressive symptoms.
Farzeneh et al. (2010)	Cross-sectional cohort study	248	ED, Iran	12–18 years	AD 84.3%; major depression 18%; personality disorder 10%	Not stated	Self-poisoning	Not stated	Not stated	Female—80.64%, childhood adversity—48%, family psychiatric history—33%, substances—11%
Galgali et al. (1998)	Retrospective study (chart review)	119	ED, India	Mean age 25 years	AD 33.7%; depression 21%; schizophrenia 4.3%	One year	Self-poisoning—most common being pesticides	9.24% of the sample had a previous attempt	Not stated/unknown	Substance abuse, epilepsy, co-morbid psychiatric illnesses
Ghimire et al. (2012)	Retrospective study (chart review)	200	ED, Nepal	15–55 and above. 77% below the age of 34 years	AD 13.5%; mood disorder 11%; substance abuse 7%	3 months	Self-poisoning by various compounds, pesticides being the most common	Not reported	Not reported	Gender, substance abuse, interpersonal conflict
Grundikoff et al. (2015)	Retrospective study (chart review)	265 93 self-harm	ED, New York	0–17 years	AD 417.7%	1 year	Not reported	57 (22.4%)	Not reported	Family conflict—30% suicidal ideation, 41% self-harm. Peer conflict—30% suicidal ideation, 41% self-harm.
Huyse et al. (2001)	Cross-sectional cohort study	1795 self-harm Total in study 10560	Liaison psychiatry services in 11 European countries	Mean age presenting with self-harm 38 years	Self-harm 17%; AD 12.4%	1 year	Not reported	Not reported	Not reported	Self-harm 56% female, 24% transferred to psychiatric ward
Lin et al. (2012)	Retrospective study (chart review)	73	Medical admissions, Taiwan	16–83 years	AD 41.1%; depression 49.3%	10 years	Charcoal burning	Not reported	Not reported	Stressors included end of relationship (18%), debt (18%) and illness (18%). Male patients had higher rates of AD, comorbid with alcohol abuse.
Lin et al. (2018)	Retrospective study (chart review)	174	Medical admissions, Taiwan	Mean 45.8 years (SD20) rodenticide group; 41.2 years (SD 14.9) paraquat group	AD *n* = 17 (9.8%)—2(3.2%) rodenticide group; 15(14.1%) paraquat group	12 years	Self-poisoning by either rodenticide or paraquat	*n* = 17 (9.8%)—3(7.6%) rodenticide group; 45(30%) paraquat group	87 (50%) total 0 rodenticide group; 87 (58%) paraquat group. No detail by diagnosis	AD significantly associated with presentation with paraquat poisoning (high lethality group)
Lingeswaren et al. (2016)	Prospective cohort study	40	Medical admissions, India	10–30 years	Acute stress reaction/Adjustment disorder in 100%	6 months	Self-poisoning	1 participant had a previous suicide attempt	Death by suicide was an exclusion criterion of this study	Female—62.5% Stressors included parenting issues 47.5%, interpersonal difficulties 30%, academic 7%
Magat et al. (2008)	Retrospective study (chart review)	65	Tertiary centre in Honolulu, Hawaii	5–18 years	AD 29%; depressive illness (45%)	2 years	Self-poisoning	26%	None	Gender (female) 86%, age 13–16 68%
McCauley et al. (2001)	Retrospective study (chart review)	70	ED, rural hospital, Ireland	10 to >60 years	AD 35.78%; depressive disorder (28.6%); schizophrenia 7.1%	1 year	92.9% overdose; 1.4% each for drowning, hanging, inhaling exhaust fumes, laceration of wrists	Absence or presence of previous suicidal behaviour is documented in 47.7% of charts.	None	Gender Female: Male 2:1, alcohol implicated in 47% of cases
Mitrev (1996)	Prospective descriptive study	140	Toxicological unit, Germany	15 to >60 years	AD 100%, no additional diagnosis	2 years	Self-poisoning	20% had a prior suicide attempt	None	Interpersonal conflict—70%, occupational/ economic—25%
Polyakova, 1998	Prospective observational study	155	ED, Moscow	18–65 years	AD 55.5%; depression 44.5%	9 months	AD group poisoning *n* = 60 (70%: males 19, 22%, female 41, 48%); hanging 17 (20%: males 12, 14%, female 5, 6%); other 9 (10%: males 4, 5%, female 5, 5%)	Not reported	None	AD less educated, lower social status, unmarried. Majority unfavourable childhood events. Alcohol 3 times more likely to be involved in AD than depression, more impulsive. AD regretted (92%, compared with only 12% in the depression group)
Suss et al. (2004)	Cross sectional cohort study	92	ED, New York	12–18 years	AD 77% of the more serious suicide attempts and 50% of the less serious suicide attempts. Other diagnoses are not listed	2 years	Self-poisoning	32% had previous suicide attempt, with 6% having two or more previous attempts	None–the study was conducted exclusively on non-fatal suicide attempts	Gender—86% of participants were female. Ethnicity 82% of participants were African American
Taggart et al. (2006)	Prospective cohort study	125 self-harm, of 167 patients in study	ED, Belfast	13–77 years	AD—49 (31.8%) clinically; 12 (7.8) SCID Depression 30 (19.5%) clinically; 56 (36.4%) SCID	1 year	Poisoning 104 (83.2%); cutting 10 (8%); other 11 (8.8%)	129 (83.8%) prior self-harm, 25 (16.4) >x2 Figures given for whole sample, figures for those presenting with self-harm not described separately	Not reported	54.5% female, 45.5% male. 67.5% previous psychiatric treatment.
Wai et al. (1999)	Retrospective study (chart review)	214	ED and medical admissions, Singapore	13–21 years	AD (53.5%), Major depression (24.3%), Schizophrenia (1.9%) Substance misuse (0.5%)	4 years	90% poisoning; 6% mixed; 4% violent incl defenestration	Not reported	Not reported	Family conflict 24.5%; conflict with friends 23.6%; school problems 11%; military service in 10% of males.
Zhargami et al. (2002)	Prospective descriptive study, included psychological autopsy	318	Burns unit, Iran	No age range given. Average age of 27 years stated	AD 42.1%; major depression 11%; anxiety d/o 4.7%; schizophrenia 4.1%	Initial interviews over 2-year period, follow up interviews 8 years later	Self-immolation	27% of cases had a previous suicide attempt	242 or 79% of the study group died as a result of self-immolation	Marital conflict (30%), family problems (12%), “love affair” (10%), conflict with spouse’s family (5%)
